# Supplementary material for: Plasmodium myosin A drives parasite invasion by an atypical force generating mechanism
Source: Nat Commun. 2019 Jul 23;10:3286. doi: 10.1038/s41467-019-11120-0 (PMC6650474; doi:10.1038/s41467-019-11120-0)
Supplement: Supplementary file 1 — Supplementary Information [file 41467_2019_11120_MOESM1_ESM.pdf]

Supplementary Information
Robert-Paganin *et al.*, 2019

Supplementary Figures

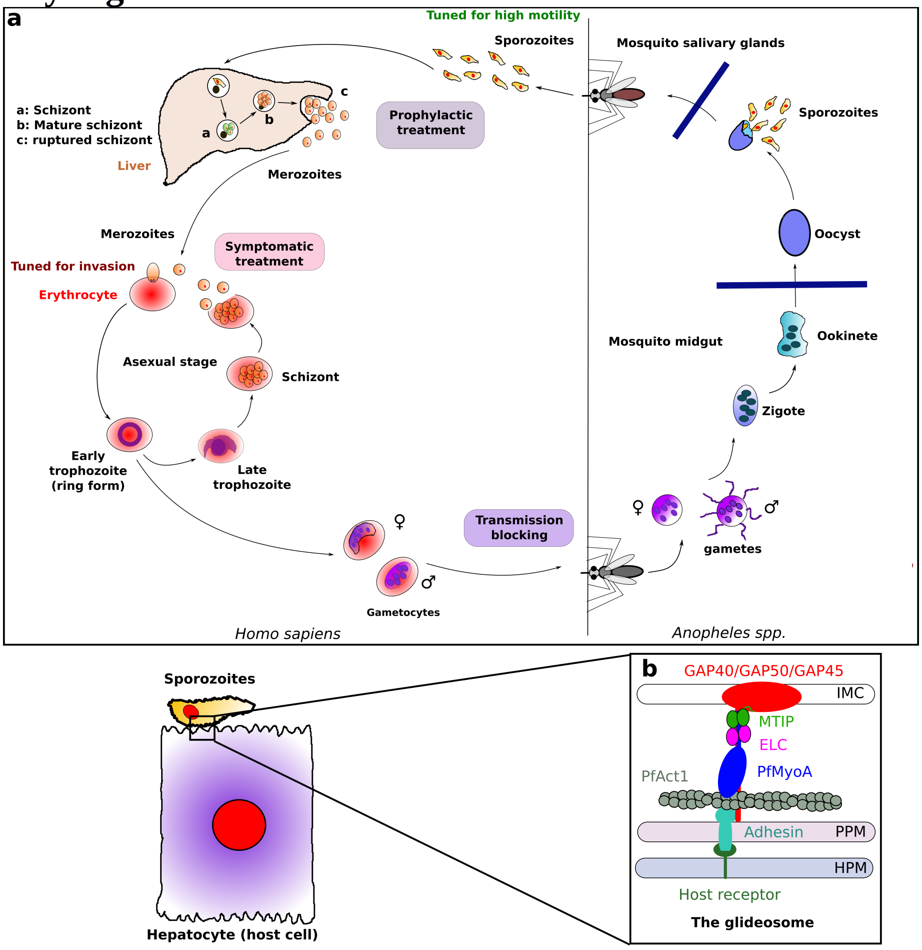

Supplementary Figure 1 | *Plasmodium falciparum* life cycle and current treatments. (a) The apicomplexan parasite *Plasmodium falciparum* displays a complex life-cycle with two hosts: mosquitos from the genus *Anopheles* and *Homo sapiens*. In the human host, the parasite alternates between highly motile stages called Sporozoites, able to use a substrate-dependent mode of locomotion broadly termed gliding motility (~2 µm/s) and immobile but invasive Merozoites. Merozoites invade blood cells, performing asexual reproduction until gametocytes appear, in order to perform sexual reproduction once they are collected via another mosquito bite. Current antimalarial therapeutics for malaria, targeting the *Plasmodium* parasite can be subdivided into three classes: **(i)** Prophylactic treatments aimed at blocking malarial infection in the early stages. Liver stage prophylaxis (Primaquine, Malarone) blocks hepatic invasion by sporozoites. Blood stage prophylaxis (Chloroquine, Mefloquine, Amodiaquine) blocks primary infection of the erythrocytes by the merozoites. These treatments are preventive but resistance by the parasite is already demonstrated[1]. **(ii)** Symptomatic treatments aim to eliminate the parasite in the asexual reproduction blood stages. The most important of these treatments are derivatives and combination therapies (ACTs) based on artemisinin which likely work in many ways such as via interfering with hemoglobin digestion by the parasite, thus blocking its development. Recent reports of resistant to ACTs suggests this frontline antimalarial is under threat. See[2] for review. **(iii)** Transmission blocking treatments which aim to block the transmission of the parasites in a population. Drugs in this class may target gametocytes or stages of development in the mosquito. For a review see[3]. **(b)** Schematic representation of the glideosome, the macromolecular complex responsible of gliding motility. Movement production relies on a specific actomyosin system comprising myosin A (PfMyoA) and actin 1 (PfAct1). PfMyoA contains the essential light chain (ELC) and the myosin tail domain interacting protein (MTIP). MTIP anchors PfMyoA to the inner membrane complex (IMC) via GAP proteins. Short and oriented PfAct1 filaments are anchored to the parasite plasma membrane (PPM) via adhesins that bind host receptors from the host plasma membrane (HPM).

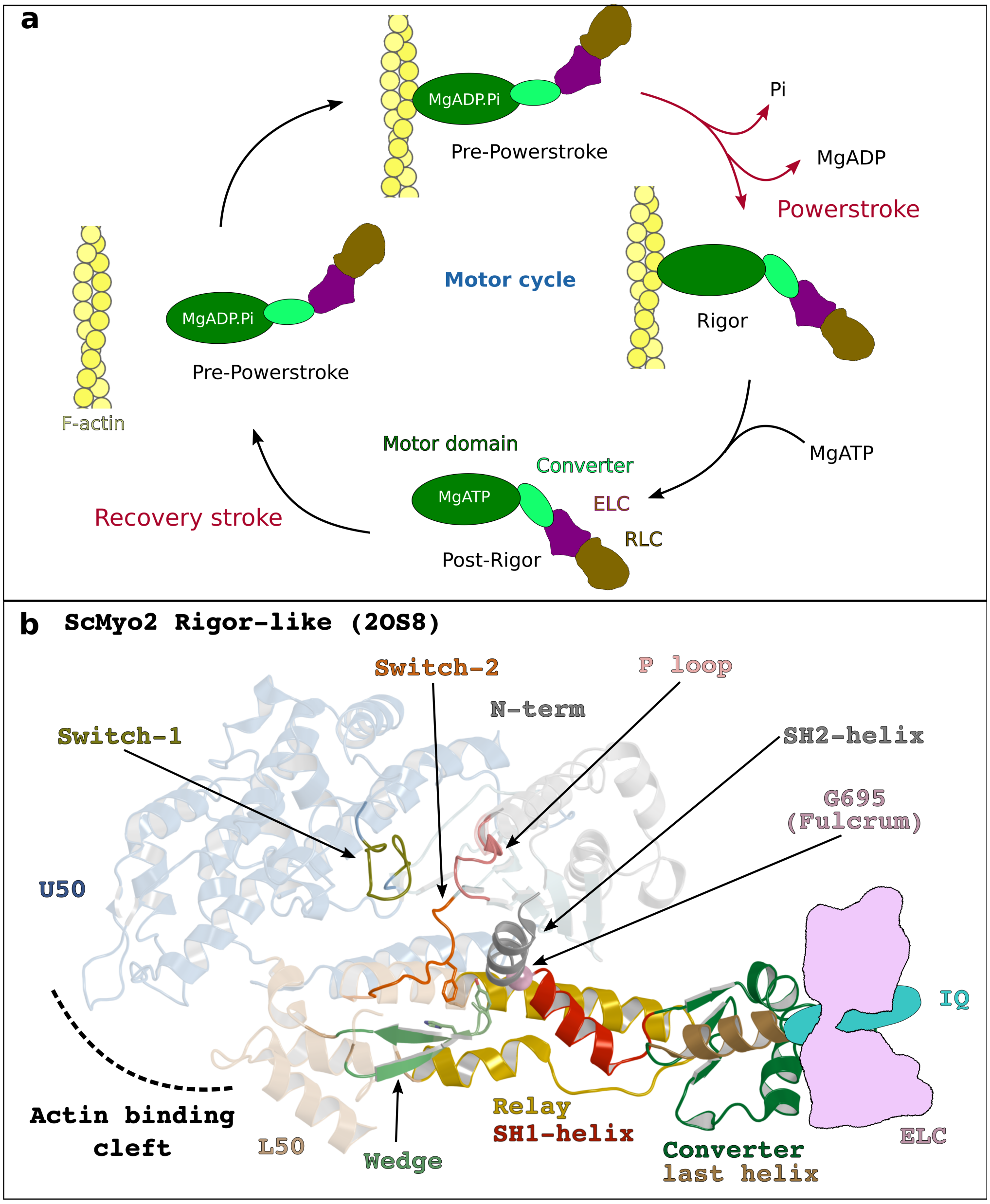

Supplementary Figure 2 | Motor cycle of a canonical myosin. (a) During the mechanical cycle, myosins explore distinct structural states depending on the nucleotide bound. ATP binding in the myosin active site triggers detachment of the motor from F-actin while myosin adopts the Post-rigor state (PR) of poor affinity for F-actin and with a lever arm down. The recovery stroke allows priming of the lever arm (comprising the essential light chain (ELC) and the regulatory light chain (RLC)), leading to the pre-powerstroke state (PPS) in which ATP hydrolysis can occur. The PPS state has a low affinity for F-actin but its association with F-actin triggers rearrangements during the powerstroke that couples the release of hydrolysis products and the swing of the lever arm. The nucleotide-free Rigor state has high affinity to F-actin. **(b)** Cartoon representation of scallop myosin II (ScMyo2) in the Rigor-like state (PDB code 2OS8). The subdomains of the myosin are shown in transparency: N-terminal (N-term, grey); Upper 50 kDa (U50); Lower 50 kDa (L50); converter (green). The myosin lever arm is composed of the converter whose last helix extends to form a helical domain containing the IQ motifs to which light chains bind. Here the first IQ domain, (cyan), which binds an ELC (pale pink) is represented schematically. The active site loops and the connectors driving the lever arm swing during the powerstroke are represented in plain: P-loop (light pink); Switch-1 (olive); Switch-2 (orange); Relay (yellow); Wedge (pale green); SH1-helix (red); SH2-helix (grey) as well as the conserved SH2-SH1 glycine (fulcrum) (pink sphere). For detail on the rearrangements of these connectors during the powerstroke see **Supplementary Fig. 4**.

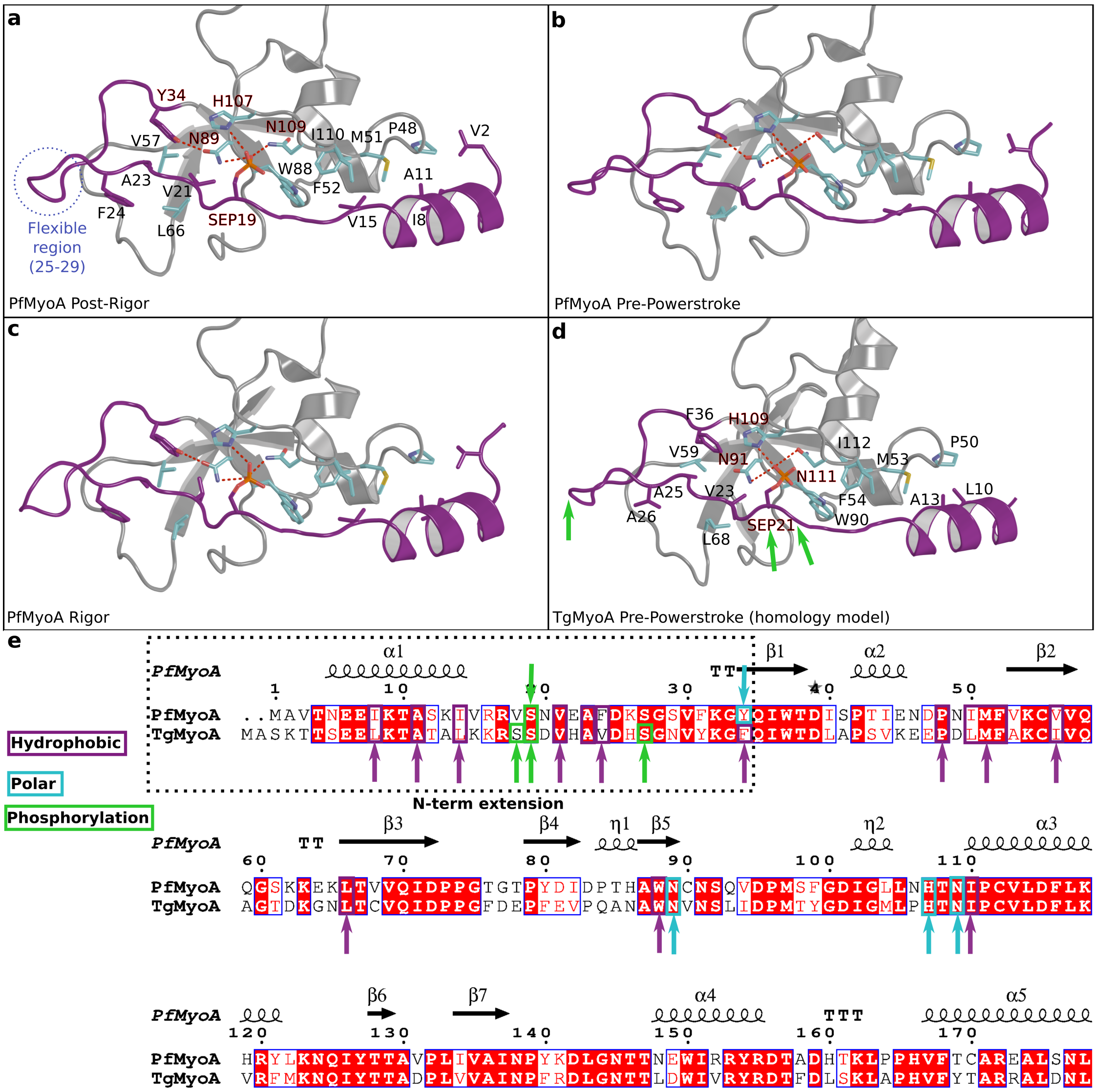

Supplementary Figure 3 | The interface between the N-term extension and the N-term subdomain is conserved during the motor cycle of PfMyoA, as well as in *T. gondii* myosin A (TgMyoA). The network of interactions between the PfMyoA N-term extension and N-term subdomain are represented in the PR state **(a)**, the PPS state **(b)** and the Rigor-like state **(c)**. In all states, these interfaces are conserved and the N-term extension stays in the same position with the same conformation, although small differences occur for the distal flexible loop (residues 25-29). Most of these interactions are hydrophobic, with an exception for the electrostatic interactions involving the phosphoserine 19 (SEP19). **(d)** A structure of the motor domain of TgMyoA lacking the N-term extension (6DUE) has been solved[4] and is similar to our PfMyoA PPS structure, but its SH3 domain is disorganized and it lacks the key N-term extension containing the phosphorylation sites. Homology modeling (SWISS-MODEL[5]) based on the Pf structures solved here predict that the TgMyoA N-term extension has the same structure and would interact similarly as shown here with the PfMyoA structures. A study of the interface between the N-term extension and the N-term subdomain of TgMyoA, suggests that the interactions described in PfMyoA are conserved in TgMyoA. This assessment is supported by the sequence alignment between the two motors. **(e)**, revealing that most of the residues involved in the interactions, including the phosphorylated serine, are conserved. Interestingly, TgMyoA displays three phosphorylation sites in the N-term extension: S20; S21 (homologous to S19 in TgMyoA) and S29. This also suggests that the tunable force-transduction mechanism described in this work may be conserved in *Toxoplasma gondii*. A complete structure of phosphorylated TgMyoA is needed to confirm these hypotheses.

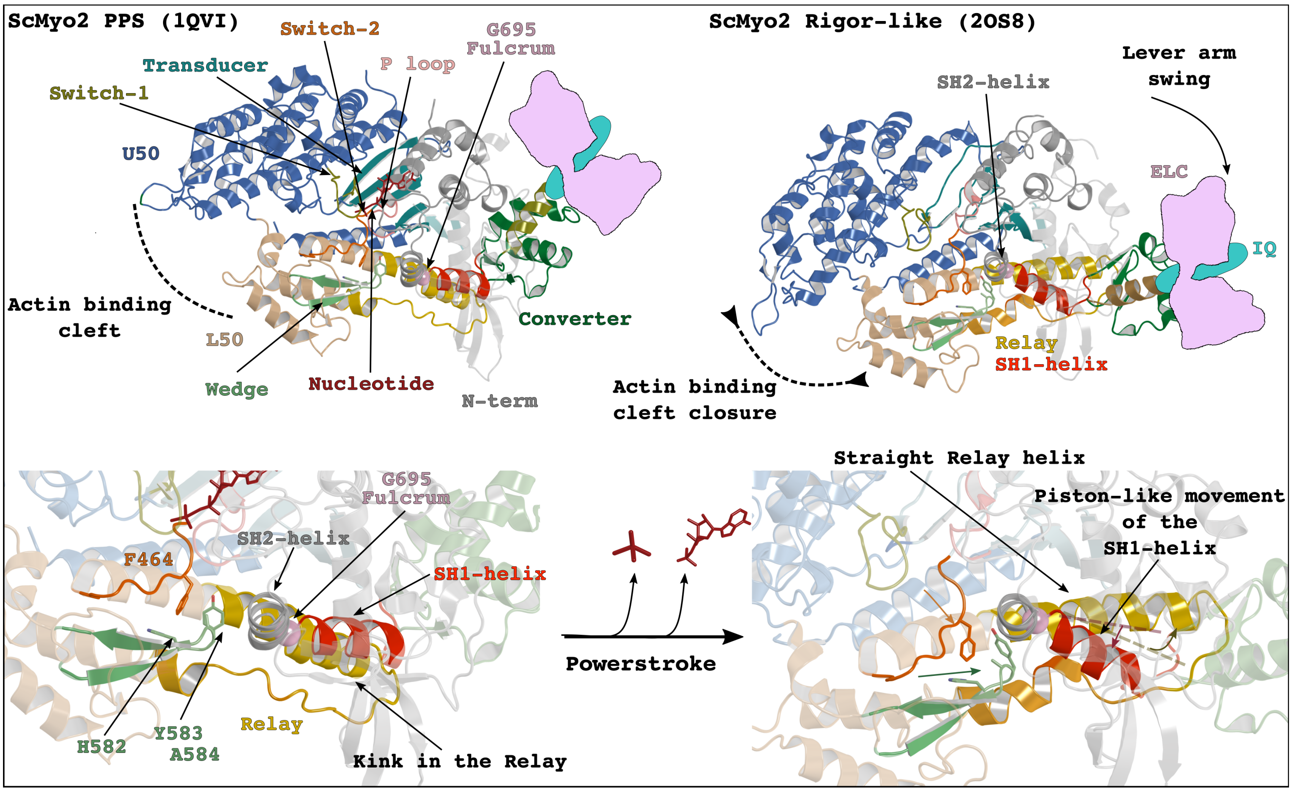

Supplementary Figure 4 | Canonical structural rearrangements in a myosin motor domain. Cartoon representation of scallop myosin II (ScMyo2) in the pre-powerstroke state (PPS) (PDB code 1QVI) and of scallop ScMyo2 in the Rigor-like state (PDB code 2OS8). (**Top**), an overall view of the motor domain is presented to show the canonical structural elements of conventional myosin. (**Bottom**), a zoom on the Wedge shows the allosteric transduction within the motor domain. During the powerstroke, sequential release of the hydrolysis products triggers global rearrangements of the motor domain. Changes in the Switch-2 conformation push the Wedge near the Relay/SH1-helix since they interact via conserved hydrophobic interactions between $^{\text{Switch-2}}$F464 and ^{Wedge}H582, ^{Wedge}Y583 and ^{Wedge}A584. The bulky side chain of ^{Wedge}Y583 participates in the release of the Relay kink and in the piston-like movement of the SH1-helix, triggering the lever arm swing.

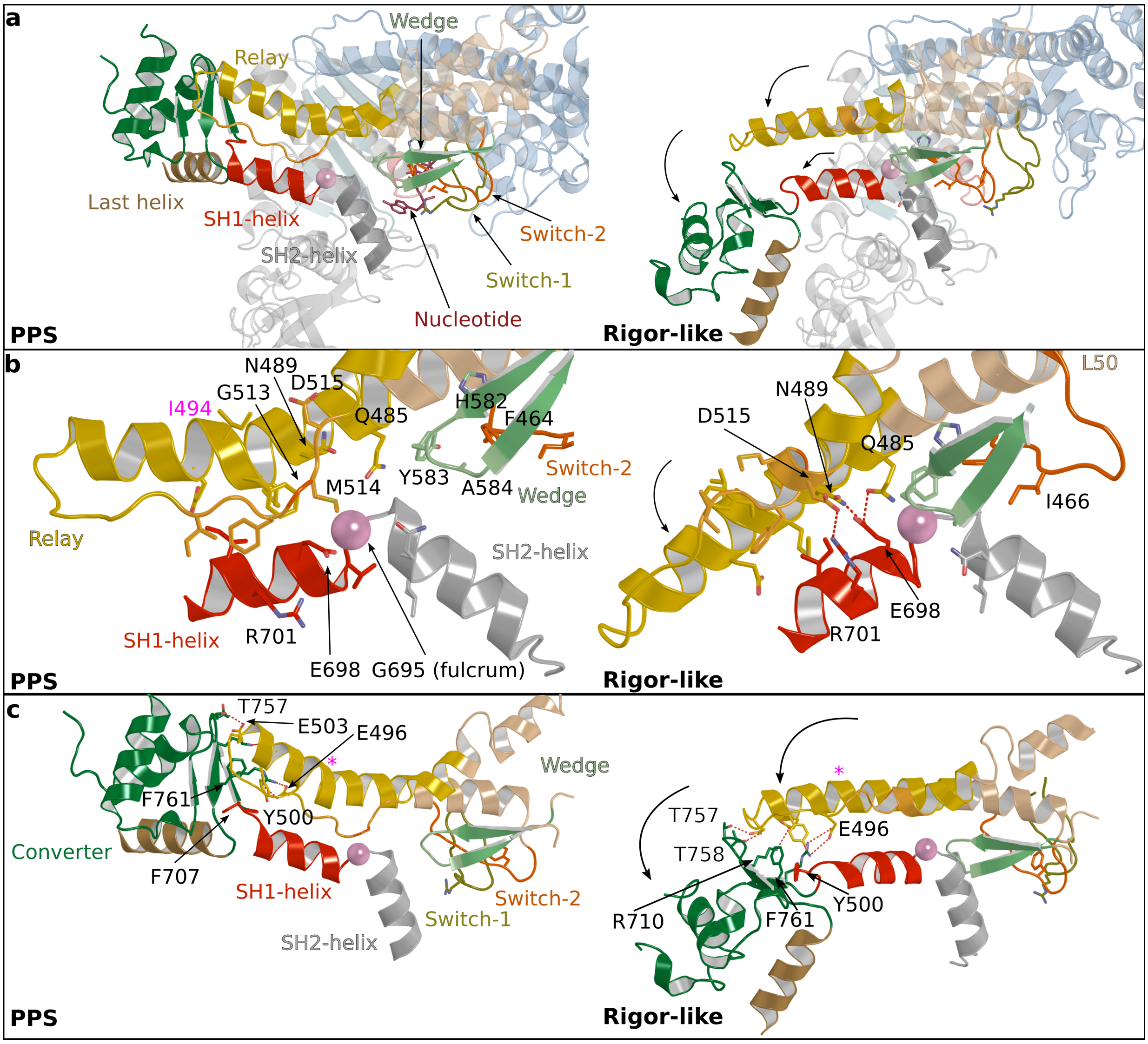

Supplementary Figure 5 | The mechanism of force production by a conventional myosin II (scallop myosin II, ScMyo2). Here the key elements in allosteric communication during the powerstroke of a conventional myosin II are presented. The structure of the pre-powerstroke state (PPS) of scallop myosin II (PDB code 1QVI) and the structure of the Rigor-like state of scallop Myo2 (PDB code 2OS8) have been used for the figures. **(a)** In the PPS state of conventional myosins, the Relay is in a primed position and it establishes a network of hydrophobic interactions with the SH1-helix. The Wedge is in a distal position from these elements. Release of hydrolysis products triggers a reorganization of Switch-2 and thus a cascade of events favoring the lever arm swing (arrows). **(b)** During the powerstroke, Switch-2 rearrangements displaces the Wedge close to the Relay and the SH1-helix. To avoid steric hindrance with the aromatic side chain of ^{Wedge}Y583, a piston-like movement of the SH1-helix occurs coordinated with the straightening of the kink in the Relay while the network of interactions between these two elements is remodeled. The piston-like movement of the SH1-helix is allowed by the presence of a deformable connector at the SH2-SH1 junction called the fulcrum (G695) (shown as a pink ball). As with PfMyoA (see Fig. 5b), in Myo2, a conserved aromatic residue from Switch-2 (F464) interacts with hydrophobic residues of the Wedge in both the PPS and Rigor-like state. This conserved interaction drives the movement of the Wedge during the powerstroke and positions it close to the Relay. The view in **(b)** is rotated ~45° anticlockwise compared to **(a)**. **(c)** Similar figure as in **(a)** in which now the major interactions between the converter and both the Relay and the SH1-helix are shown. These interactions transmit and amplify the structural rearrangements of the motor domain. Thus, the piston-like movement of the SH1-helix and the straightening of the Relay kink trigger the swing of the converter.

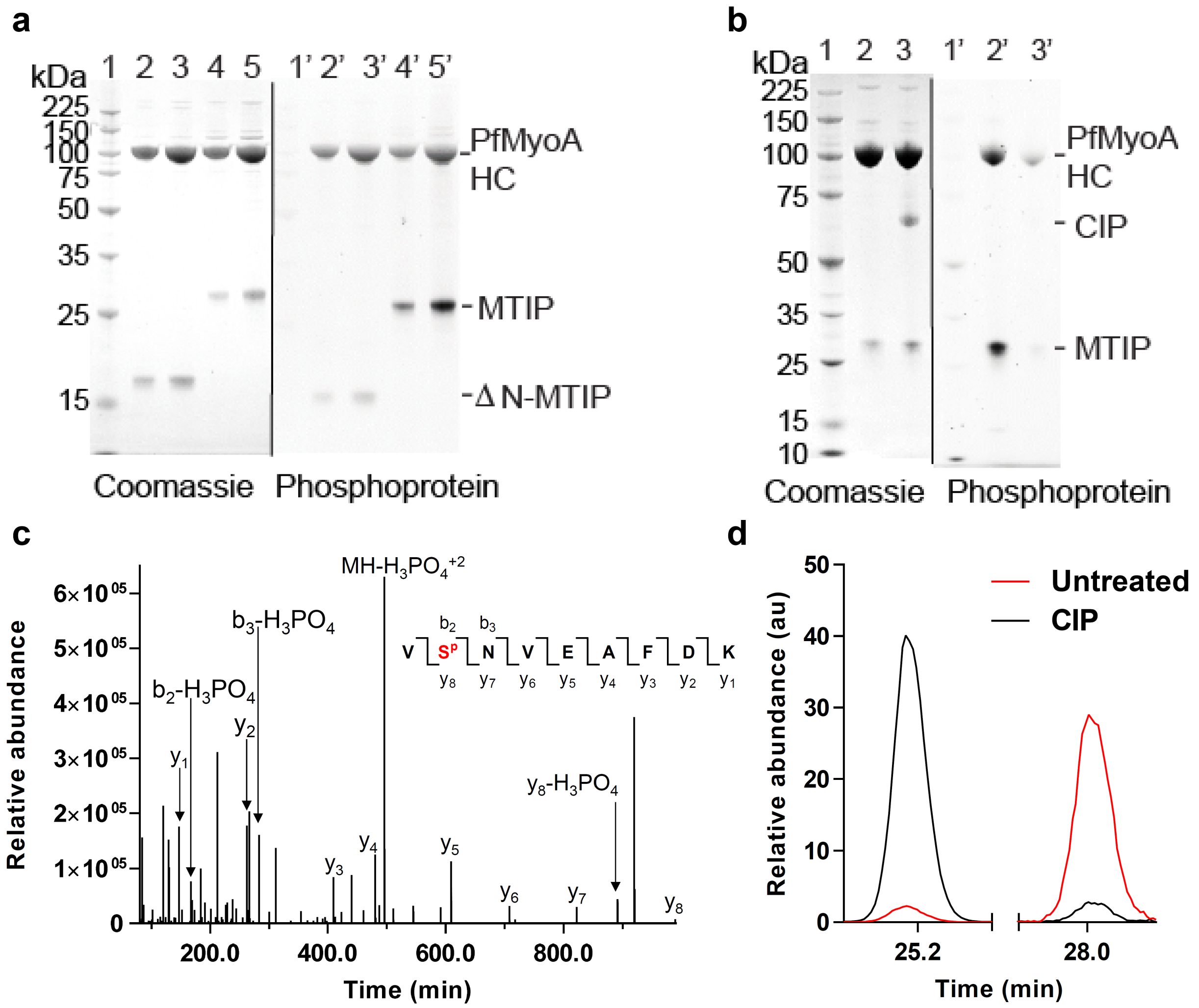

Supplementary Figure 6 | Gel and mass spectrometry-based analyses of PfMyoA phosphorylation state. (a) 12% SDS-PAGE of PfMyoA expressed with full-length MTIP or truncated ΔN-MTIP (starts at residue Ser61), stained with Coomassie (left panel) or ProQ Diamond (Invitrogen, P33300) phosphoprotein stain (right panel). Lanes 1,1', molecular mass markers; lanes 2, 2', 3, 3', two loads of PfMyoA-ΔN-MTIP; lanes 4, 4',5, 5', two loads of PfMyoA-MTIP. The heavy chain and full-length MTIP were phosphorylated, but ΔN-MTIP was not. **(b)** 4-12% SDS-PAGE of PfMyoA expressed with full-length MTIP and stained with Coomassie (left panel) or ProQ Diamond phosphoprotein stain (right panel). Lanes 1, 1', molecular mass markers; lanes 2, 2' PfMyoA as expressed in *Sf9* cells; lanes 3, 3' following dephosphorylation with calf intestinal phosphatase (CIP; New England Biolabs, M0290S). Both the heavy chain and MTIP were dephosphorylated by CIP. **(c)** PfMyoA bands were excised from the 4-12% SDS-PAGE gels, digested with trypsin to produce peptides and analyzed by liquid chromatography mass spectrometry (LCMS). LCMS MS fragmentation spectra of the VSpNVEAFDK phosphopeptide containing PfMyoA serine 19. The sequence identity and position of phosphate were confirmed by the continuum of fragment ions (y- and b-ions). Similar mass spec analyses identified the sites of phosphorylation on MTIP as Ser 51, 55, 58 and 61[6]. **(d)** LCMS peptide elution profiles for the non-phosphorylated VSNVEAFDK (RT = 25.2 min; m/z = 504.7535) and phosphorylated VSpNVEAFDK (RT = 28.0 min; m/z = 544.7366) peptides found in the untreated and CIP-treated PfMyoA samples. Y-axis for the right panel was increased 40-fold to account for the difference in the intrinsic ionization efficiency between the non-phosphorylated and phosphopeptides (right panel). 96 ± 1% (± SD, n = 6, 2 independent preparations) of the peptides found in the untreated sample were phosphorylated at serine 19 and CIP treatment removed almost all of the phosphate from the peptide.

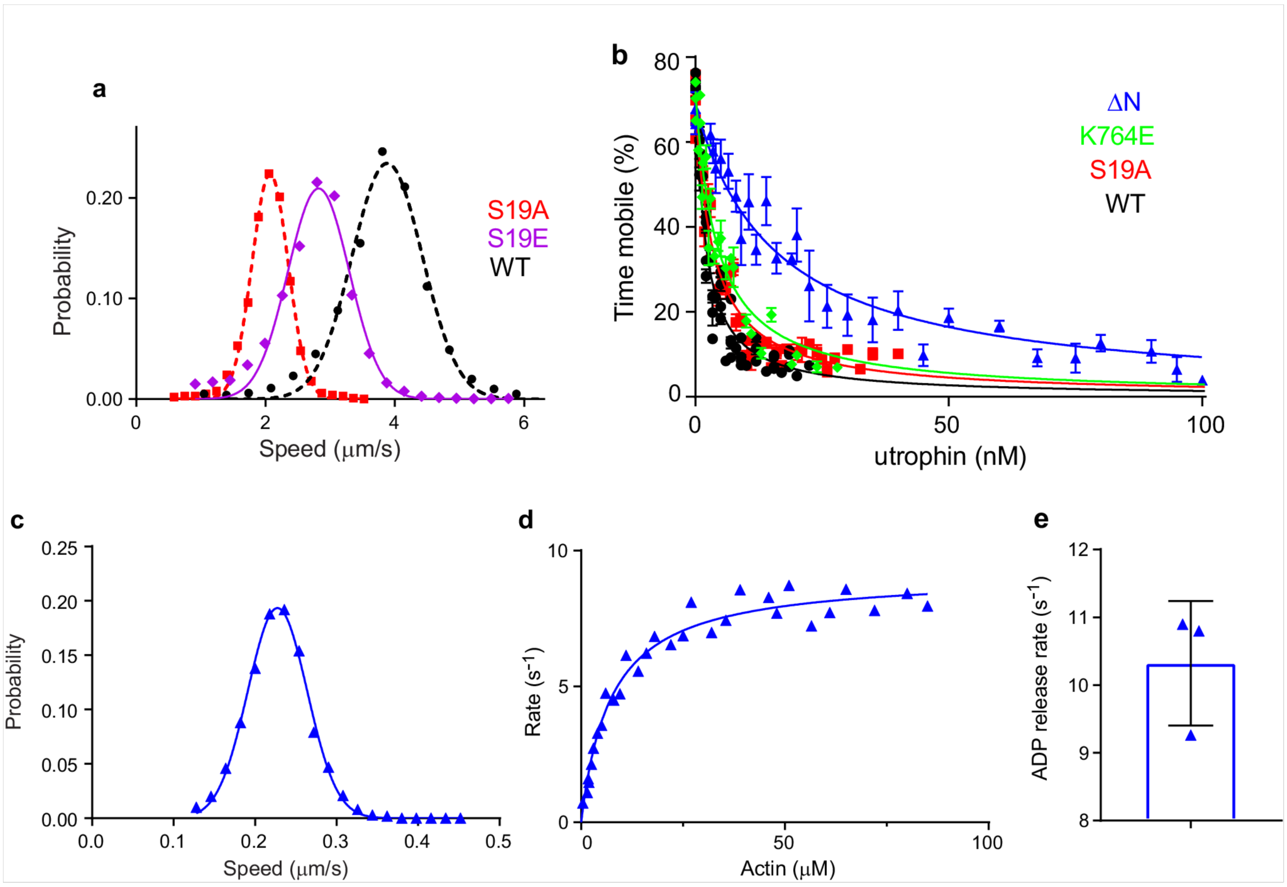

Supplementary Figure 7 | Phospho-mimic S19E does not recapitulate phosphorylation, ensemble force data shown in Fig. 5e extended to higher utrophin concentrations, and data for ΔN with expanded y-axis scale compared with Fig. 5b-d. (a) Speed distributions from a representative *in vitro* motility assay showing that the phospho-mimic S19E does not recapitulate phosphorylation. WT (black), 3.88 ± 0.54 μm/sec (n=4294 filaments); S19A (red), 2.07 ± 0.28 μm/sec (n=5291 filaments); S19E (purple), 2.82 ± 0.47 μm/sec (n=4213 filaments). Values are mean ± SD. **(b)** Ensemble force fits from Fig. 5e with data at higher utrophin concentrations shown. **(c)** *In vitro* motility speed for **ΔN**, **(d)** actin-activated ATPase activity for **ΔN**, and **(e)** ADP release rate for **ΔN**, each with expanded y-axis scales compared with Fig. 5b-d. Source data are provided as Source Data File.

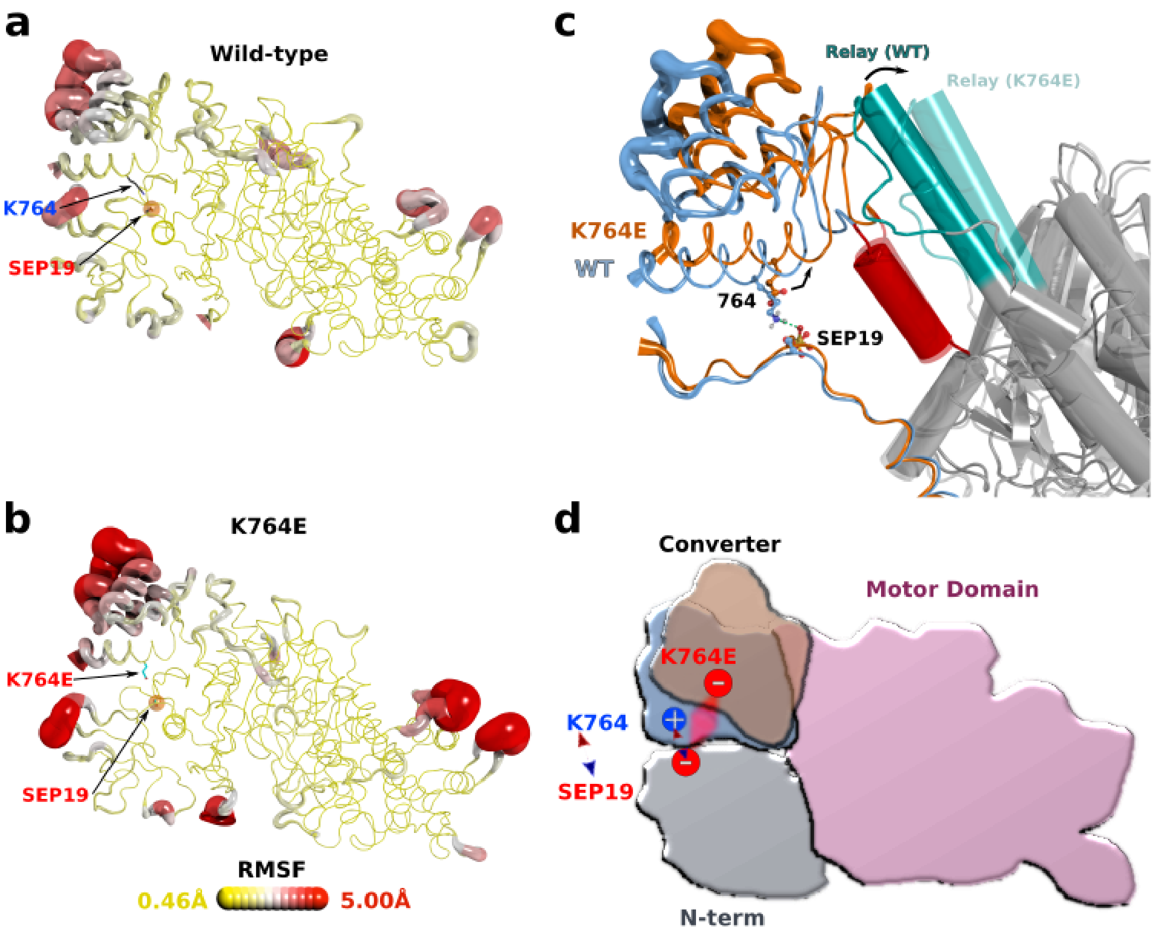

Supplementary Figure 8 | Molecular dynamics of the phosphorylated wild-type (WT) and K764E mutant. The mutant K764E in the Rigor state has been studied *in silico* to analyze the effect of introducing a repulsive interaction instead of the electrostatic bond between ^{Converter}K764 and SEP19 from the N-term extension. On the left (**a** and **b**), a "putty representation" of the PfMyoA motor domain in the Rigor state (aa 1-768) represents the RMS fluctuations observed during 60 ns simulations with RMS scale ranging from 0.6 Å (yellow) to 4.8 Å (red). **(a)** WT, **(b)** K764E. In **(c)**, superimposition on the N-term subdomain of the two putty representations. In the WT, during the entire duration of the dynamics, the converter stays in position and the electrostatic bond established between SEP19 and K764 is maintained. In contrast, the electrostatic repulsion introduced in the K764E mutant fails to maintain the converter in position. It is progressively displaced from its original position together with the Relay but not with the SH1-helix that maintains its position. **(d)** Schematic representation of the molecular dynamics results: the motor domain is represented in pale purple, the N-term in light grey, the WT converter in light blue and the K764E converter in light orange. In the WT, the converter establishes an electrostatic bond with SEP19 from the N-term extension via K764, this stabilizes the Rigor-like conformation of PfMyoA. When the mutation K764E is introduced, the converter is no longer stabilized in its rigor conformation and is progressively exploring positions that are further away from the N-terminal subdomain and its WT position.

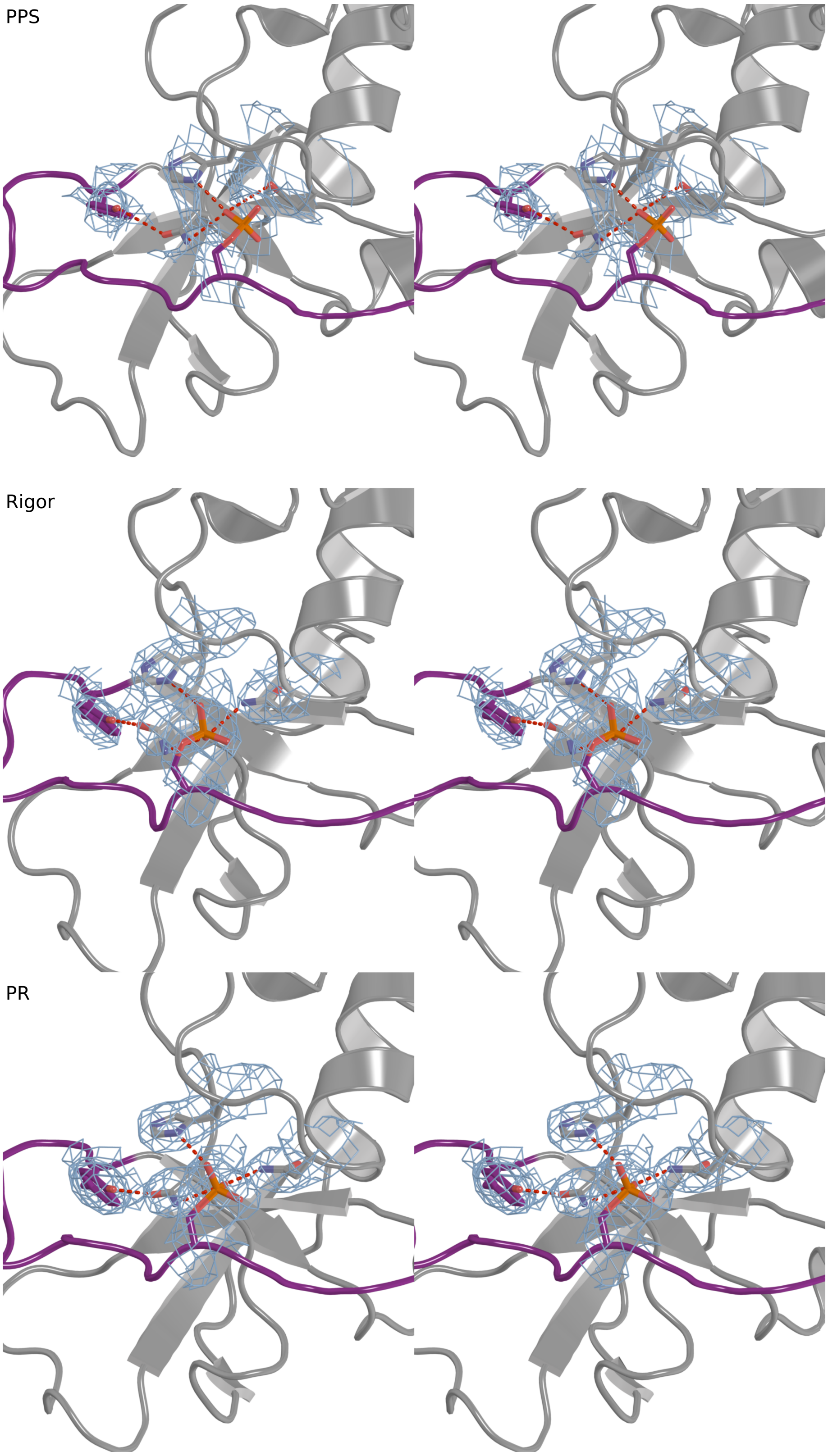

Supplementary Figure 9 | Stereo view of the electron-density map for the phosphoserine SEP19 in the three PfMyoA structural states. The 2Fo-Fc electron density map contoured at 1.0 σ is presented for each state. The structures of the Rigor and the Post-rigor (PR) states comes from the asymmetric unit of the same crystal (crystal type 1) and have been solved at a resolution of 2.82 Å. The structure of the pre-powerstroke state (PPS) has been solved at 3.49 Å (crystal type 2). Despite the lower resolution for the PPS state, the electron density map is well-defined for SEP19 and for the interacting residues. See **Supplementary Tables 1 & 2** for data processing and refinement statistics.

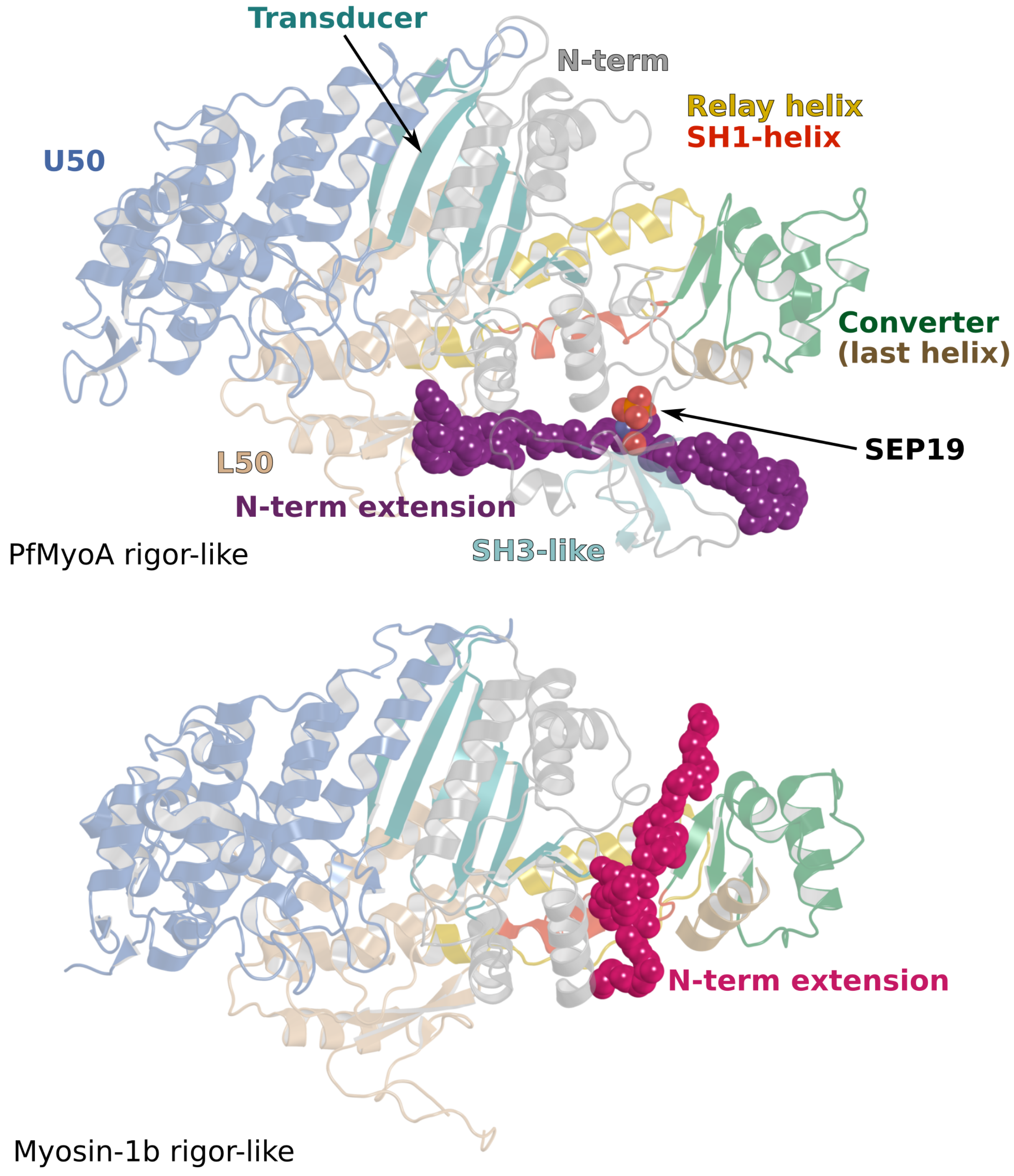

Supplementary Figure 10 | N-terminus extension in PfMyoA and Myosin-1b. (a) Cartoon representation of the Rigor-like state of PfMyoA (top) and of Myosin-1b (Myo1b) (bottom, PDB code 4L79). The N-term extension of PfMyoA and Myo1b are represented as spheres and colored in purple and hotpink respectively. The comparison between the two structures illustrates the differences in terms of location and orientation between the N-term extensions of these two myosins. Note that Class 1 myosins do not have a SH3-like domain as part of their N-term sequence as most other class do (shown in cyan for PfMyoA). Color code: N-term (grey), U50 (marine blue), L50 (wheat tint), Relay helix (yellow), SH1-helix (red), transducer (deepteal cyan), converter (green), last helix of the converter (yellow sand).

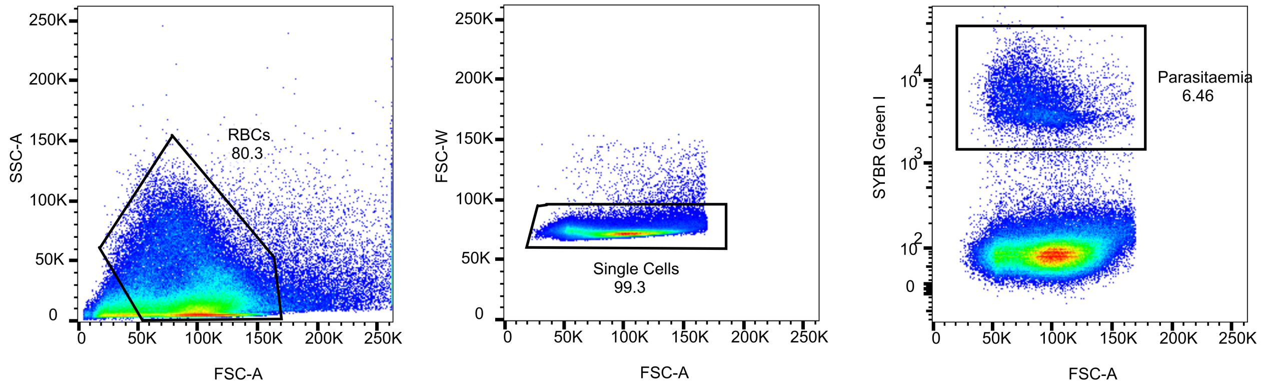

Supplementary Figure 11 | flow cytometry plots showing the gating strategy used to produce data for Fig 1B. RBCs were gated out, then single cells, then infected RBCs as DNA-positive cells.

Supplementary Tables

Supplementary Table 1. Data collection and refinement statistics of crystal type 1 (Rigor-like and PR states). PDB code 6I7D.

	**Rigor-like and PR MD PfMyoA**
**Data collection**	
Space group	$P2_1$
Cell dimensions	
a, *b*, *c* (Å)	63.52, 258.73, 103.09
α, β, γ (°)	90 92.08 90
Resolution (Å)	44.22-2.82 (2.921-2.82)*
R_{meas}	0.1163 (0.8961)
$I / \sigma I$	12.54 (1.97)
$CC_{1/2}$ (%)	99.7 (70.5)
Completeness (%)	98.75 (96.41)
Redundancy	5.1 (5.0)
**Refinement**	
Resolution (Å)	44.22-2.82
No. reflections	403,952 (total), 78757 (unique)
R_{work} / R_{free} (%)	18.70/24.10
No. atoms	
Protein	23,917
Ligand/ion	14
Water	423
B-factors	
Protein	71.85
Ligand/ion	57.10
Water	53.64
R.m.s. deviations	
Bond lengths (Å)	0.015
Bond angles (°)	1.82

*Values in parentheses are for highest-resolution shell.

Supplementary Table 2. Data collection and refinement statistics of crystal type 2 (PPS state). PDB code 6I7E.

	**PPS MD PfMyoA**
**Data collection**	
Space group	$P6_1$ 2 2
Cell dimensions	
a, *b*, *c* (Å)	197.460, 197.460, 175.144
α, β, γ (°)	90 90 120
Resolution (Å)	171.005-3.492 (3.735-3.492)*
R_{meas} (all I+ and I-)	0.186 (2.456)
R_{meas} (within I+/I-)	0.201 (2.490)
$I / \sigma I$	12.10 (1.40)
$CC_{1/2}$ (%)	99.9 (69.4)
Completeness (%) (Spherical)	59.5 (16.50)#
Completeness (%) (Ellipsoidal)	94.7 (66.4) #
Redundancy	22.6 (21.8)#
**Refinement**	
Resolution (Å)	25.17-3.492 (3.615-3.492)
No. reflections	31114 (total), 15557 (unique)
R_{work} / R_{free} (%)	19.50/23.30
No. atoms	
Protein	6061
Ligand/ion	33
Water	0
B-factors	
Protein	150.76
Ligand/ion	140.47
Water	
R.m.s. deviations	
Bond lengths (Å)	0.017
Bond angles (°)	1.88

*Values in parentheses are for highest-resolution shell.
Data collection values obtained after using StarAniso.

Supplementary Table 3. ***In vitro*** **motility speed distributions of various PfMyoA constructs.**

**PfMyoA construct**	**mean (µm/s)**	**±SD (µm/s)**	**Number of Filaments**
WT	3.84	0.55	4408
	3.92	0.53	5475
	3.82	0.59	3748
	3.90	0.47	6600
	3.89	0.66	3634
S19A	2.07	0.33	6441
	2.04	0.35	6072
	1.99	0.26	5039
	2.02	0.26	5250
	2.07	0.28	5260
K764E	1.76	0.29	3962
	1.70	0.24	5555
	1.73	0.32	3079
	1.75	0.23	5962
ΔN	0.23	0.04	2310
	0.26	0.04	3485
	0.25	0.03	2474
	0.23	0.03	1696
	0.22	0.04	2046

WT, 5 technical replicates, 3 protein preparations; S19A, 5 technical replicates, 2 protein preparations; K764E, 4 technical replicates, 2 protein preparations; ΔN, 5 technical replicates, 2 protein preparations. Conditions: 25 mM imidazole, pH 7.5, 150 mM KCl, 1 mM EGTA, 4 mM $MgCl_2$, 10 mM DTT, 2 mM MgATP, 0.5% (w/v) methylcellulose, 25 µg/ml PfELC, 25 µg/ml PfMTIP and oxygen scavengers (50 µg/ml catalase, 125 µg/ml glucose oxidase, and 3 mg/ml glucose. 30°C. SD, standard deviation.

Supplementary Table 4. Rate of ADP release from actomyosin at various temperatures.

PfMyoA construct	20°C (s^{-1})	25°C (s^{-1})	30°C (s^{-1})
WT	79.4 ± 0.6	171.5 ± 0.9	285.9 ± 2.3
	98.0 ± 0.5	188.2 ± 1.2	320.4 ± 2.5
	131.4 ± 1.2	266.0 ± 2.2	384.4 ± 6.0
	93.1 ± 0.7	186.2 ± 1.2	333.2 ± 3.1
	114.2 ± 1.0	233.1 ± 3.4	347.8 ± 4.8
S19A	30.2 ± 0.1	-	111.5 ± 0.2
	29.8 ± 0.1	58.4 ± 0.2	107.7 ± 0.3
	39.1 ± 0.1	72.9 ± 0.3	128.2 ± 0.6
K764E	18.1 ± 0.1	43.2 ± 0.2	95.2 ± 0.6
	26.8 ± 0.1	56.2 ± 0.3	112.3 ±0.9
	20.2 ± 0.2	45.1 ± 0.4	103.0 ± 1.2
ΔN	1.64 ± 0.01	3.78 ± 0.01	9.26 ± 0.01
	2.10 ± 0.01	5.03 ± 0.01	10.90 ± 0.01
	1.99 ± 0.01	4.79 ± 0.01	10.80 ± 0.02

Data from 3-5 protein preparations are shown for each construct. Conditions: 10 mM HEPES, pH 7.5, 50 mM KCl, 4 mM $MgCl_2$, 1 mM EGTA, and 1 mM DTT.

References

1. Schwartz, E. Prophylaxis of malaria. *Mediterr. J. Hematol. Infect. Dis.* **4**, e2012045–e2012045 (2012).
2. Martin, R. E., Shafik, S. H. & Richards, S. N. Mechanisms of resistance to the partner drugs of artemisinin in the malaria parasite. *Curr. Opin. Pharmacol.* **42**, 71–80 (2018).
3. Sinden, R. E. Targeting the Parasite to Suppress Malaria Transmission. *Adv. Parasitol.* **97**, 147–185 (2017).
4. Powell, C. J. *et al.* Structural and mechanistic insights into the function of the unconventional class XIV myosin MyoA from Toxoplasma gondii. *Proc. Natl. Acad. Sci. U. S. A.* **115**, E10548–E10555 (2018).
5. Waterhouse, A. *et al.* SWISS-MODEL: homology modelling of protein structures and complexes. *Nucleic Acids Res.* **46**, W296–W303 (2018).
6. Bookwalter, C. S. *et al.* Reconstitution of the core of the malaria parasite glideosome with recombinant Plasmodium class XIV myosin A and Plasmodium actin. *J. Biol. Chem.* **292**, 19290–19303 (2017).
