# Supplementary material for: Plasmodium myosin A drives parasite invasion by an atypical force generating mechanism
Source: Nat Commun. 2019 Jul 23;10:3286. doi: 10.1038/s41467-019-11120-0 (PMC6650474; doi:10.1038/s41467-019-11120-0)
Supplement: Supplementary file 3 — Reporting Summary [file 41467_2019_11120_MOESM3_ESM.pdf]

natureresearch

Corresponding author(s): Anne Houdusse (anne.houdusse@curie.fr)

Last updated by author(s): Jun 9, 2019

Reporting Summary

Nature Research wishes to improve the reproducibility of the work that we publish. This form provides structure for consistency and transparency in reporting. For further information on Nature Research policies, see Authors & Referees and the Editorial Policy Checklist.

Statistics

For all statistical analyses, confirm that the following items are present in the figure legend, table legend, main text, or Methods section.

n/a	Confirmed	
☐	☒	The exact sample size (*n*) for each experimental group/condition, given as a discrete number and unit of measurement
☐	☒	A statement on whether measurements were taken from distinct samples or whether the same sample was measured repeatedly
☐	☒	The statistical test(s) used AND whether they are one- or two-sided<br>*Only common tests should be described solely by name; describe more complex techniques in the Methods section.*
☒	☐	A description of all covariates tested
☒	☐	A description of any assumptions or corrections, such as tests of normality and adjustment for multiple comparisons
☐	☒	A full description of the statistical parameters including central tendency (e.g. means) or other basic estimates (e.g. regression coefficient) AND variation (e.g. standard deviation) or associated estimates of uncertainty (e.g. confidence intervals)
☐	☒	For null hypothesis testing, the test statistic (e.g. *F*, *t*, *r*) with confidence intervals, effect sizes, degrees of freedom and *P* value noted<br>*Give P values as exact values whenever suitable.*
☒	☐	For Bayesian analysis, information on the choice of priors and Markov chain Monte Carlo settings
☒	☐	For hierarchical and complex designs, identification of the appropriate level for tests and full reporting of outcomes
☒	☐	Estimates of effect sizes (e.g. Cohen's *d*, Pearson's *r*), indicating how they were calculated

Our web collection on statistics for biologists contains articles on many of the points above.

Software and code

Policy information about availability of computer code

Data collection	Flow cytometry data collected in FACSDiva (BD). IFA images collected in NIS-Elements (Nikon). DNA gel imaged with Image Lab (Bio-Rad).
Data analysis	Flow cytometry data analysed in FlowJo. IFA images processed in Icy and ImageJ2. Prism GraphPad v7; RRID:SCR_002798 was used to graph and perform statistical analysis on data in Fig. 5a-e and Supplementary Fig 7a-e. Speed data in Fig. 5b,e and Supplementary Figure 7a-c were analyzed by Fast Automated Spud Trekker v1.1 (FAST), which is available for free download at spudlab.stanford.edu (also see Aksel et al., 2015 DOI:10.1016/j.celrep.2015.04.006)

For manuscripts utilizing custom algorithms or software that are central to the research but not yet described in published literature, software must be made available to editors/reviewers. We strongly encourage code deposition in a community repository (e.g. GitHub). See the Nature Research guidelines for submitting code & software for further information.

Data

Policy information about availability of data

All manuscripts must include a data availability statement. This statement should provide the following information, where applicable:

- Accession codes, unique identifiers, or web links for publicly available datasets
- A list of figures that have associated raw data
- A description of any restrictions on data availability

A data availability statement has been added to the manuscript. The two crystallographic structures discussed in this work have been deposited to the PDB (PDB codes 6I7D and 6I7E).

Field-specific reporting

Please select the one below that is the best fit for your research. If you are not sure, read the appropriate sections before making your selection.

☒ Life sciences ☐ Behavioural & social sciences ☐ Ecological, evolutionary & environmental sciences

For a reference copy of the document with all sections, see nature.com/documents/nr-reporting-summary-flat.pdf

Life sciences study design

All studies must disclose on these points even when the disclosure is negative.

Sample size	Each growth assay measured parasitemia in 100,000 red blood cells, which is sufficient to accurately measure parasitemias at these levels. Two independent clones of the MyoA-cKO line were tested to rule out anomalous effects.
Data exclusions	No data were excluded.
Replication	For Fig 1B, three separate biological replicates were performed for each of the cell lines, sufficient given the effect size is far greater than the margin of error between the experiments (with the exception of the heparin negative control where two replicates were judged sufficient since the effect of heparin is well established). The genotyping (Fig 1C) and western blot (Fig 1E) were repeated for each of the replicates of the growth assay, with a representative result being shown. The GFP band in the western blot (Fig 1E) could not always be detected due to a low level of expression of the truncated protein, but the consistent absence of the FLAG band and growth phenotype meant the effects were clear.
Randomization	Red blood cells from the same donor were used in each replicate. All the infected cultures were treated the same during each replicate.
Blinding	Blinding was not carried out.

Reporting for specific materials, systems and methods

We require information from authors about some types of materials, experimental systems and methods used in many studies. Here, indicate whether each material, system or method listed is relevant to your study. If you are not sure if a list item applies to your research, read the appropriate section before selecting a response.

Materials & experimental systems

n/a	Involved in the study	
☐	☒	Antibodies
☐	☒	Eukaryotic cell lines
☒	☐	Palaeontology
☒	☐	Animals and other organisms
☒	☐	Human research participants
☒	☐	Clinical data

Methods

n/a	Involved in the study	
☒	☐	ChIP-seq
☐	☒	Flow cytometry
☒	☐	MRI-based neuroimaging

Antibodies

Antibodies used	anti-FLAG (Sigma, F1804, clone M2). anti-GFP (Roche, 11814460001, clones 7.1/13.1). anti-PfAct1 (PMID: 22389687). anti-GAP45 (PMID: 16321976)
Validation	anti-FLAG validated by manufacturer ("The monoclonal antibody detects only the target protein band(s) on a Western blot from an E. coli, plant or mammalian crude cell lysate"). anti-GFP validated by manufacturer ("Anti-GFP is tested for functionality and purity relative to a reference standard to confirm the quality of each new reagent preparation"). anti-PfAct1 validated in PMID: 22389687. anti-GAP45 validated in PMID: 16321976.

Eukaryotic cell lines

Policy information about cell lines

Cell line source(s)	B11 Plasmodium falciparum obtained from M. Treeck, Crick Institute (PMID 29970464)
Authentication	Cell lines were not authenticated.
Mycoplasma contamination	Cell lines were not tested for mycoplasma.

Commonly misidentified lines (See ICLAC register)	None used.

Flow Cytometry

Plots

Confirm that:

☒ The axis labels state the marker and fluorochrome used (e.g. CD4-FITC).

☒ The axis scales are clearly visible. Include numbers along axes only for bottom left plot of group (a 'group' is an analysis of identical markers).

☒ All plots are contour plots with outliers or pseudocolor plots.

☒ A numerical value for number of cells or percentage (with statistics) is provided.

Methodology

Sample preparation	Human RBCs from donors were infected with Plasmodium falciparum parasites. Samples were stained with SYBR Green I and washed.
Instrument	BD LSRFortessa
Software	Cytometry data was collected in FACSDiva (BD) and analysed in FlowJo.
Cell population abundance	Samples consisted of uninfected RBCs and infected RBCs (2-10% of total) based on SYBR Green I staining presence/absence of DNA
Gating strategy	FSC vs SSC was used to select RBCs (excluding very small and very large particles). FSC-A vs FSC-W was used to select singlets. FSC-A vs SYBR Green I used to select DNA-positive RBCs (i.e. parasite infected), which formed a distinct positive population, as compared to RBCs-only and RBCs+parasites+inhibitor negative controls.

☒ Tick this box to confirm that a figure exemplifying the gating strategy is provided in the Supplementary Information.
