# Supplementary material for: Plasmodium myosin A drives parasite invasion by an atypical force generating mechanism
Source: Nat Commun. 2019 Jul 23;10:3286. doi: 10.1038/s41467-019-11120-0 (PMC6650474; doi:10.1038/s41467-019-11120-0)
Supplement: Supplementary file 4 — Description of Additional Supplementary Files [file 41467_2019_11120_MOESM4_ESM.pdf]

Supplementary Movies

Supplementary Movie 1 | Comparison between ScMyo2 and PfMyoA PPS and Rigor-like. This movie allows the comparison between PfMyoA and scallop myosin 2 (ScMyo2). The comparison is made for two states: the pre-powerstroke (PPS, top) and the Rigor-like (bottom). The myosin subdomains are colored distinctly: the N-terminus extension (Nterm extension, purple), the N-terminus (grey), the upper 50 kDa (marine blue), the lower 50 kDa (wheat tint), the converter (green ; last helix in smudge green), the Relay helix (yellow), the SH1-helix (red). The beta strands and the loop of the Wedge are colored in dark green and pale green respectively. The fulcrum is represented as a ball in light pink. Interactions stabilizing the Rigor-like conformation in PfMyoA are shown with dashed circles.

Supplementary Movie 2 | Allosteric communication within the motor domain during the powerstroke. This movie allows the comparison between ScMyo2 (top) and PfMyoA (bottom). In both motors, the sequential release of the hydrolysis products (Pi and MgADP) triggers rearrangements, in particular in the connectors such as Switch-2. The movement of the Switch-2 pushes the Wedge close to the Relay/SH1-helix region. In all conventional myosins such as ScMyo2, a conserved aromatic from the Wedge creates steric hindrance that causes the straightening of the Relay kink as well as the piston-like movement of the SH1-helix, accompanying the swing of the converter. In PfMyoA, sequence adaptations within the Wedge and at the SH2-SH1 junction result in a mostly immobile SH1-helix. The converter swing is thus triggered by the Relay alone. The N-term extension (in purple) establishes additional contacts that help the stabilization of the Rigor conformation, compensating the lack of mobility of the SH1-helix: (i) phosphoserine 19 (SEP19) establishes an electrostatic bond with ^{Converter}K764, stabilizing the Rigor position of the converter and (ii) $^{\text{N-term.extension}}$Glu6 establishes a salt bridge-π interaction with $^{\text{Switch-1}}$Arg241 and $^{\text{Switch-2}}$Phe476. For further details, see **Figure 4**, **Supplementary Figures 2 & 3** and **Supplementary Movies 3 & 4**. The color code of the myosin subdomains is the same as that used in **Supplementary Movie 1**. The side chains of some key residues are shown in the two myosins: the residue from the Wedge ($_{\text{ScMyo2}}$Y583); the SH1-helix $_{\text{ScMyo2}}$E698 in dark red, the Relay $_{\text{ScMyo2}}$G513 & $_{\text{ScMyo2}}$M514 in smudge green.

Supplementary Movie 3 | Conformational changes occurring near the Relay/SH1-helix region during the powerstroke. This movie allows the comparison between ScMyo2 (top) and PfMyoA (bottom). In both motors, the Wedge triggers the straightening of the Relay helix. In ScMyo2, the piston-like movement of the SH1-helix helps the straightening of the Relay, while in PfMyoA the SH1-helix stays mostly immobile. For further details on the sequence adaptations of PfMyoA in this region see **Figure 3 & 4**, **Supplementary Figure 5** and **Supplementary Movie 2**. The color codes for the subdomains and the reporter residues are identical to **Figures 2 & 4**.

Supplementary Movie 4 | Sequence adaptations and steric hindrance caused by the Wedge during the powerstroke. This movie shows the transition between the PPS and the Rigor states of PfMyoA wild-type (WT) (top) and PfMyoA carrying a mutation replacing T586 by a tyrosine (bottom). A major sequence adaptation in PfMyoA is the presence of a threonine (T586) in the wedge instead of an aromatic. This threonine is fully adapted to create a moderate steric hindrance with the Relay which also displays sequence adaptations. Importantly, this sequence adaptation also fits better to S691 (which corresponds to the fulcrum glycine in other myosins) at the SH2-SH1 junction. The *in silico* modelling of a tyrosine (as found in canonical Myo2) at the T586 location (bottom panel) shows how the tyrosine side chain would cause a steric clash (purple star) in Rigor and collide S691.

Supplementary Movie 5 | Molecular dynamics of PfMyoA wild-type on a duration of 60 ns. The N-term domain is colored in dark grey, the converter in pink and the rest of the motor domain in dark blue. In the simulation of the WT, the converter is maintained in the Rigor-like position by the electrostatic bond between the phosphoserine 19 (SEP19) from the N-term extension and the lysine K764 from the converter during all the duration of the experiment.

Supplementary Movie 6 | Molecular dynamics of PfMyoA K764E on a duration of 60 ns. The N-term domain is colored in dark grey, the converter in pink and the rest of the motor domain in dark blue. In the simulation of the mutant K764E, the converter progressively fluctuates from the Rigor-like position due to electrostatic repulsion.
