# Supplementary figures and images for: Plasmodium myosin A drives parasite invasion by an atypical force generating mechanism
Source: Nat Commun. 2019 Jul 23;10:3286. doi: 10.1038/s41467-019-11120-0 (PMC6650474; doi:10.1038/s41467-019-11120-0)

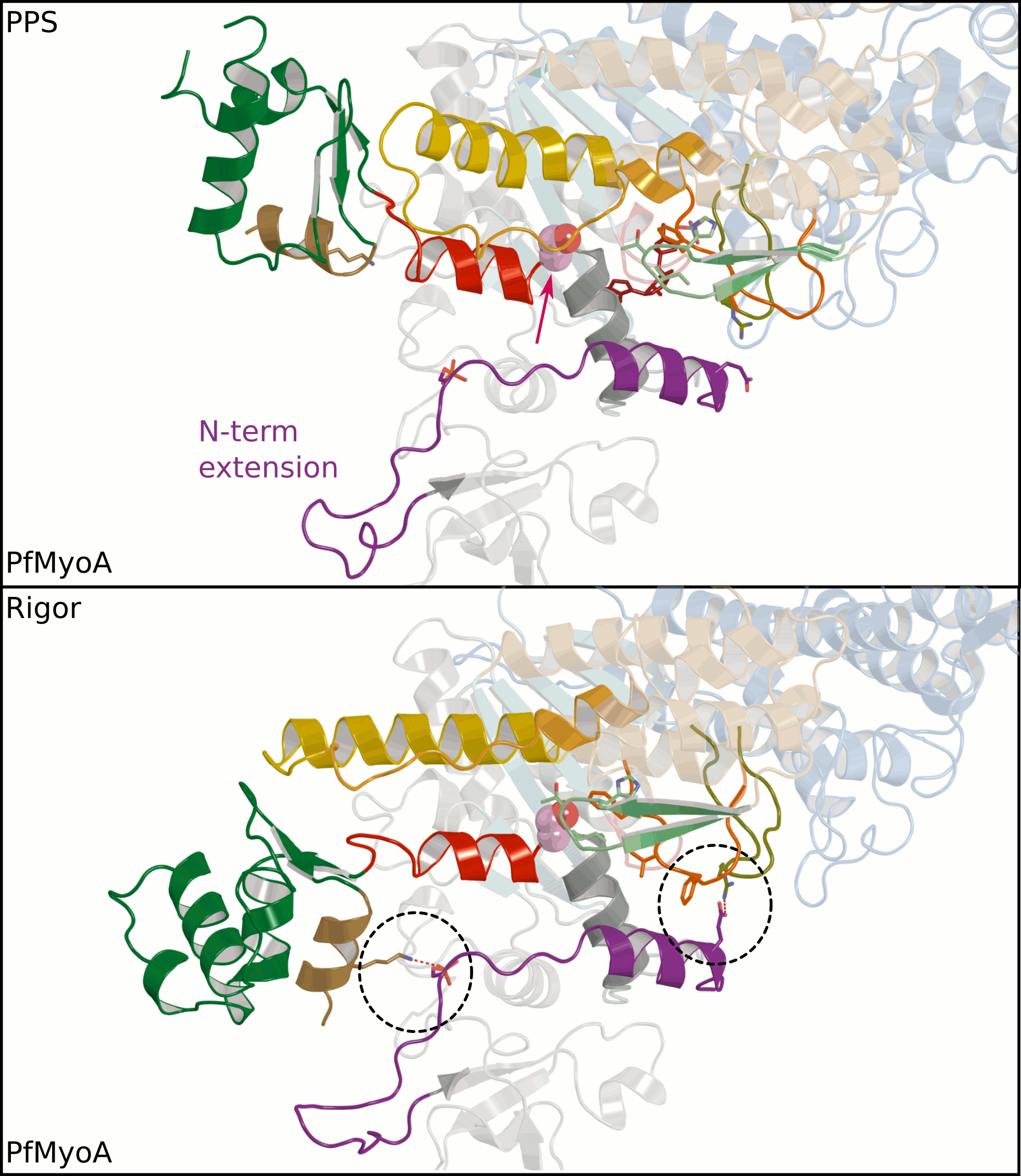

Supplement: Supplementary file 5 — Supplementary Movie 1 [file 41467_2019_11120_MOESM5_ESM.gif]

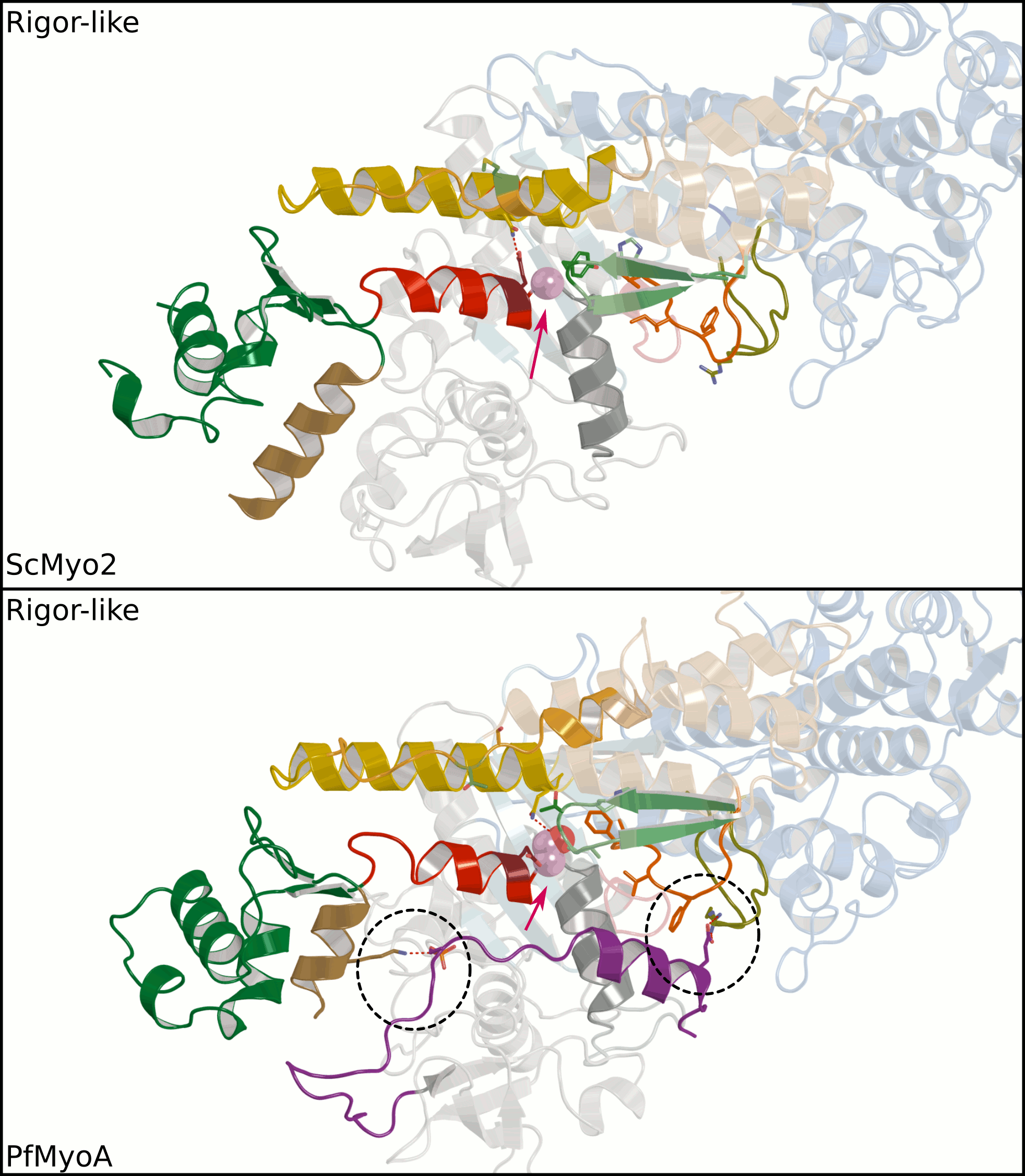

Supplement: Supplementary file 6 — Supplementary Movie 2 [file 41467_2019_11120_MOESM6_ESM.gif]

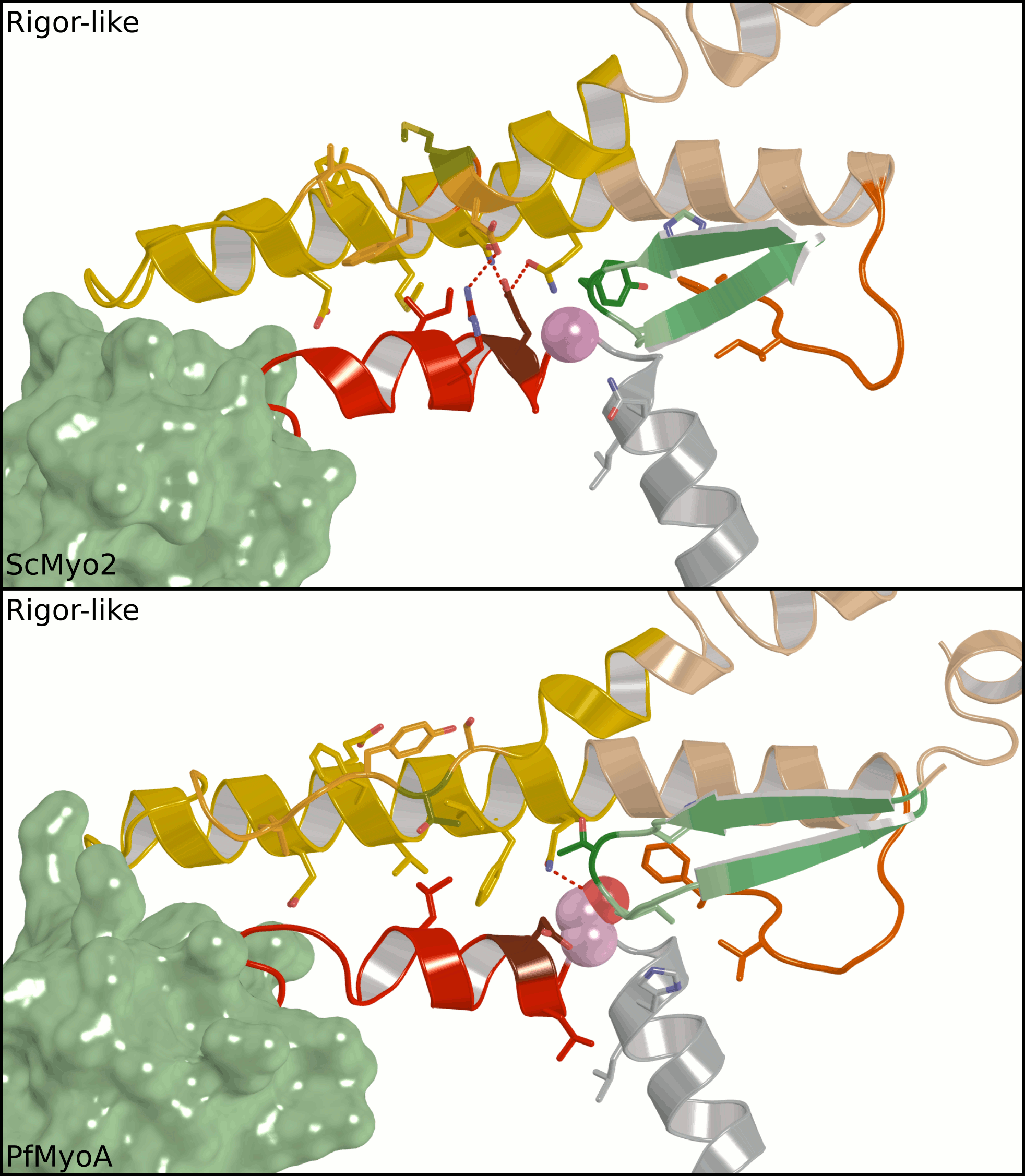

Supplement: Supplementary file 7 — Supplementary Movie 3 [file 41467_2019_11120_MOESM7_ESM.gif]

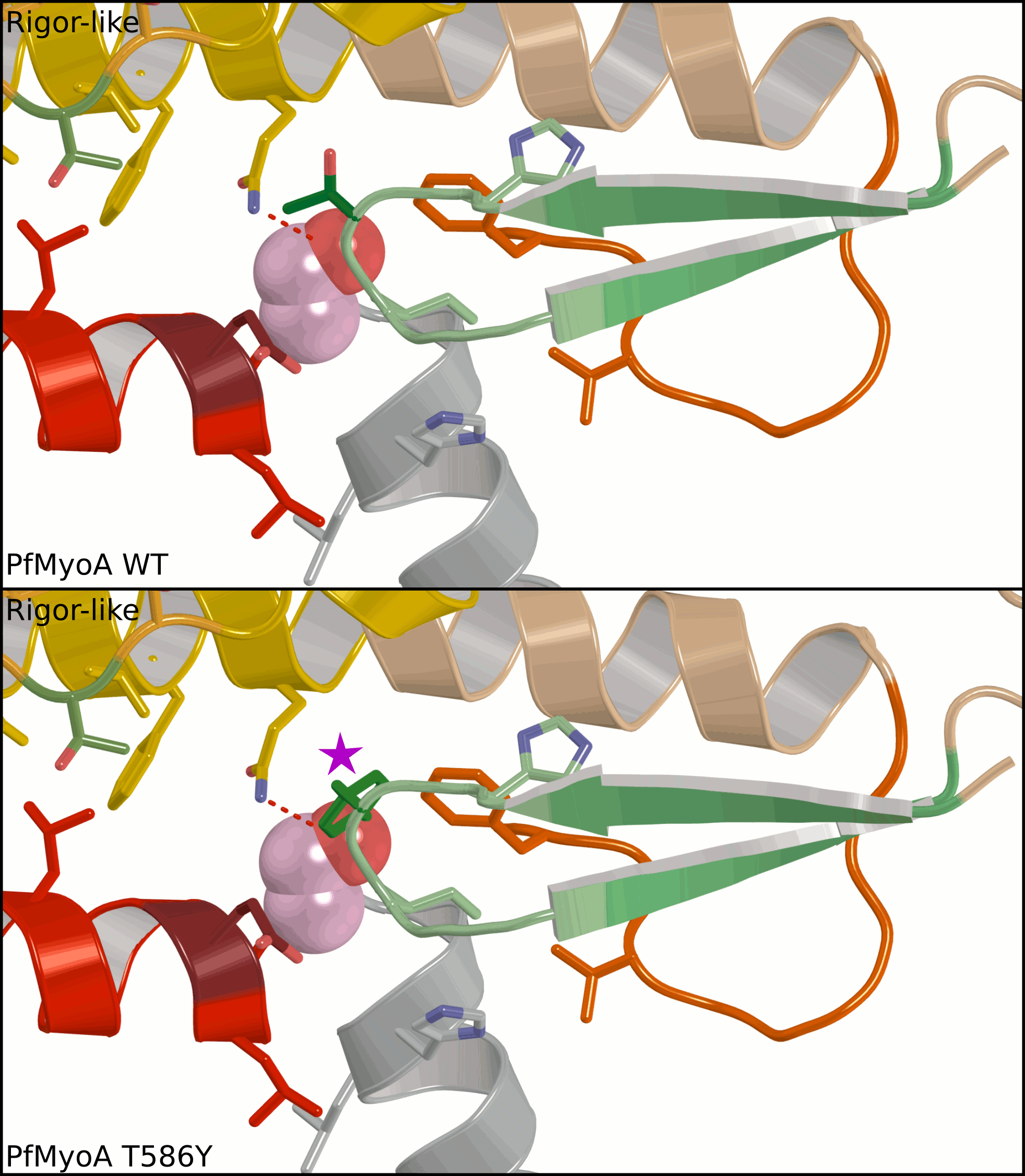

Supplement: Supplementary file 8 — Supplementary Movie 4 [file 41467_2019_11120_MOESM8_ESM.gif]
